# Supplementary material for: The interactome of intact mitochondria by cross-linking mass spectrometry provides evidence for coexisting respiratory supercomplexes
Source: Mol Cell Proteomics. 2017 Dec 8;17(2):216–32. doi: 10.1074/mcp.RA117.000470 (PMC5795388; doi:10.1074/mcp.RA117.000470)
Supplement: Supplemental Data [file supp_RA117.000470_133922_0_supp_23978_fzffwf.zip › spectra_annotation/mito_spectra_annotation/47-1-8-1-10-1.pdf]

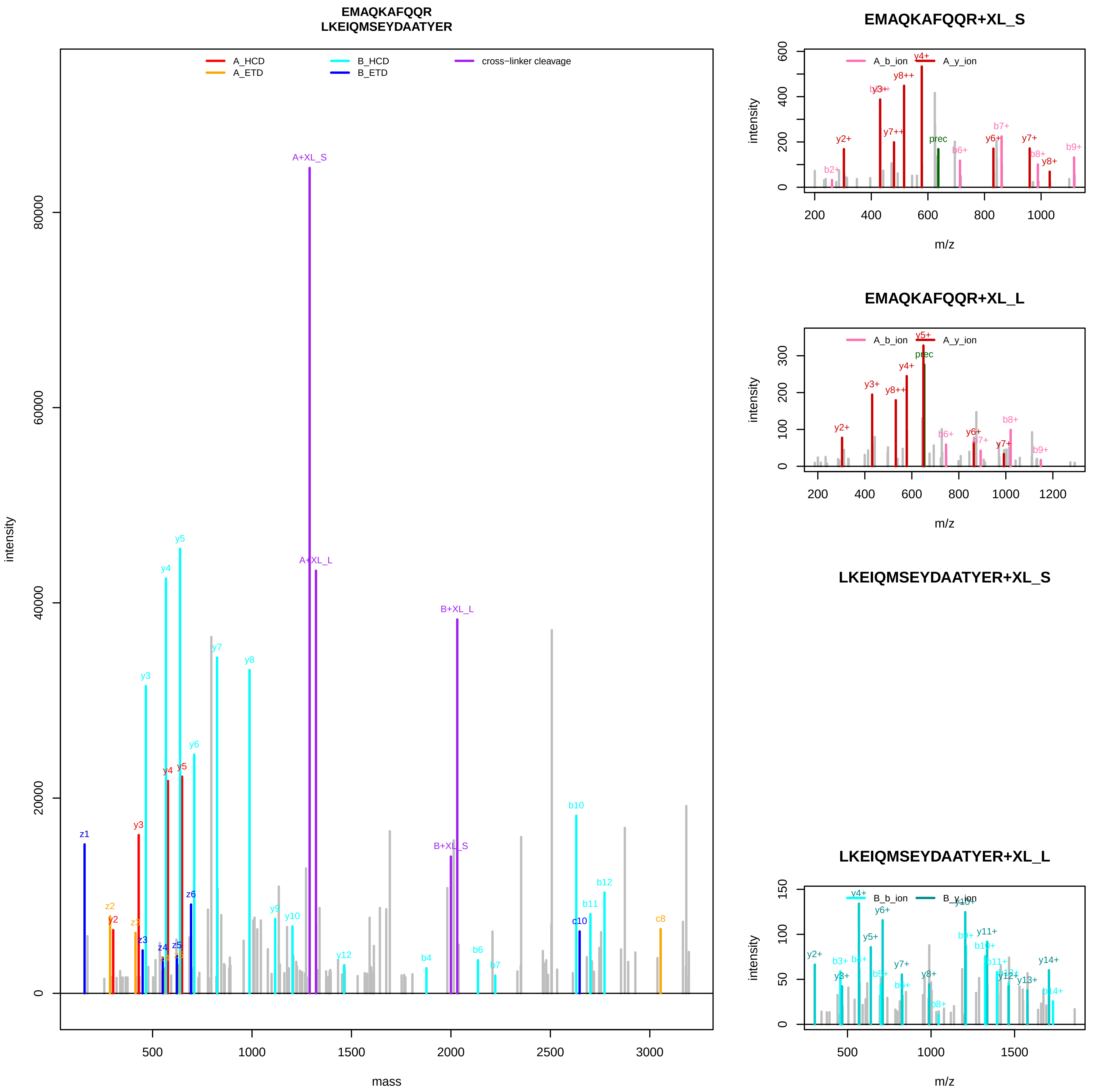
EMAQKAFQQR
LKEIQMSEYDAATYER
A_HCD
A_ETD
B_HCD
B_ETD
cross−linker cleavage
A+XL_S
A+XL_L
B+XL_L
B+XL_S
z1
z2
y2
z3
y3
z4
z5
z6
y3
y4
y5
y4
y5
y6
y7
y8
y9
y10
y12
b4
b6
b7
c10
b10
b11
b12
c8
intensity
mass
0
20000
40000
60000
80000
500
1000
1500
2000
2500
3000
EMAQKAFQQR+XL_S
A_b_ion
A_y_ion
y4+
y8++
y3+
y7++
y2+
b2+
prec
b6+
b7+
y6+
y7+
b8+
y8+
b9+
intensity
m/z
0
200
400
600
800
1000
EMAQKAFQQR+XL_L
A_b_ion
A_y_ion
y5+
prec
y4+
y3+
y8++
y2+
b6+
y6+
b7+
b8+
y7+
b9+
intensity
m/z
0
100
200
300
1200
LKEIQMSEYDAATYER+XL_S
LKEIQMSEYDAATYER+XL_L
B_b_ion
B_y_ion
y2+
y3+
b3+
y4+
y5+
y6+
b5+
y7+
b6+
y8+
b8+
b9+
y10+
b10+
y11+
b11+
y12+
y13+
y14+
b14+
intensity
m/z
0
50
100
150
500
1000
1500
